# Heavy metal accumulation and food safety of lettuce (*Lactuca sativa* L.) amended by bioslurry and chemical fertilizer application

**DOI:** 10.1002/fsn3.4363

**Published:** 2024-07-25

**Authors:** Tsigereda Meskelu, Abate Feyissa Senbeta, Yadessa Gonfa Keneni, Getachew Sime

**Affiliations:** ^1^ Department of Biology Hawassa University Hawassa Ethiopia; ^2^ Center for Ethiopian Rift Valley Studies Hawassa University Hawassa Ethiopia

**Keywords:** bioslurry, food quality, heavy metals, human health, soil health, toxicity

## Abstract

The accumulation of heavy metals in soil and plant tissue is a serious concern since it impacts both soil quality and food safety. This study evaluated the accumulation of heavy metals and the food quality of lettuce as a result of the application of chemical fertilizer (CF) and bioslurry (BS). The treatments were CF (158 kg ha^−1^ NPS and 200 kg ha^−1^ urea), BS (5 ton ha^−1^), and control with no fertilizer, laid out in a randomized complete block design with three replications. Soil samples were analyzed for their physico‐chemical characteristics. The concentrations of 10 heavy metals (As, Pb, Zn, Cd, Cu, Ni, Co, Fe, Mn, and Cr) in the agricultural soil, bioslurry, and lettuce tissue were determined. Both the BS and CF reduced the concentrations of most heavy metals in the agricultural soil, particularly As, Pb, and Cd. Only the mean concentration of Cd in the agricultural soils exceeded the threshold level set by WHO/FAO (2011) for agricultural soils. Similarly, the concentration of As, Pb, and Cd in lettuce tissue grown in BS‐treated soils and the concentration of As in agricultural soils surpassed the limit set for vegetables. Given the toxicities of As, Cd, and Pb, as well as the effect on food safety, human health, and the environment, it is unsafe to cultivate lettuce using either the agricultural soil or BS in the study area.

## Graphical abstract

Application of bioslurry reduced the concentration of most of the heavy metals in the control soil, including As, Pb, and Cd. Application of chemical fertilizers increased the bioavailability of As, Pb, and Cd for uptake by lettuce. Application of bioslurry further amplified the Cd concentration in plant tissues and its impacts on food safety and human and environmental health.

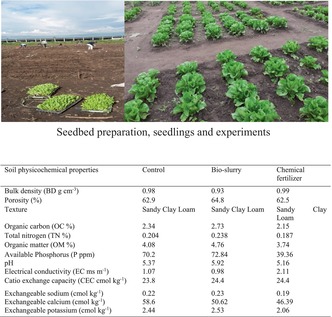

## 1 INTRODUCTION

Globally, lettuce (*Lactuca sativa* L.) is among the most familiar salad crops and occupies an extensive cultivation area. It is cultivated in many countries and extensively grown in home gardens (Madar & Hájos, 2021; Mengistu et al., 2021), requires a short growing season, low production costs, and is rich in nutrients with low calories (Faran et al., 2023). Besides, lettuce grows in diverse kinds of soils and climatic conditions and favors silt loams and sandy soils. Several lettuce cultivars are available that are simple to grow, productive in small areas, and almost completely free of diseases and pests (Tekle et al., 2018; Zemichael et al., 2017).

Vegetables can be grown using different kinds of organic fertilizers. Growers often use organic fertilizers, which might have negative impacts on the environment and food system. Bioslurry is an organic fertilizer that is carbon‐rich (Wang et al., 2023). It is also rich in phosphorous (P), nitrogen (N), potassium (K), and organic matter, which improve the physicochemical properties of the soil. Among the range of soil properties improved are porosity, water‐holding capacity, soil bulk density, soil pH, and nutrient retention. It has been used as a soil fertility enhancer in nutrient‐deficient and acidic tropical and subtropical soils (Musse et al., 2020). Therefore, the application of bioslurry could be an available scenario to boost soil fertility and enhance agricultural productivity (Alberdi et al., 2018; Wang et al., 2023).

The use of organic fertilizers might have negative consequences, despite the fact that they increase productivity and improve soil fertility. Organic fertilizers could contain high levels of harmful elements and might be the source of heavy metal contamination in the soil and crops (Liu et al., 2023; Zhen et al., 2020). In this regard, assurance of food safety – that food will not cause harm to consumer – is important when it is prepared and/or consumed (WHO/FAO, 2011). For example, the long half‐lives, high bioaccumulation potential, and non‐biodegradable nature of heavy metals resulting from fertilizer application make fresh vegetables extremely harmful to human health (Bigdeli & Seilsepour, 2008). Heavy metal contamination of soil is a frequent occurrence and can be a significant source of metals for crops, which may eventually enter the food chain and expose humans to these potentially harmful metals (Ahmad et al., 2019). According to earlier studies, heavy metals are significant contaminants in vegetables (Bhardwaj et al., 2023; Gebeyehu & Bayissa, 2020; Yeshiwas, 2017). This demonstrates unequivocally that vegetables are much more vulnerable to soil contamination by elevated levels of heavy metals (Sandeep et al., 2019). Excessive levels of heavy metals and vital trace elements like zinc and copper can build up in the human body and cause serious health issues (Khan et al., 2016). Because clearing contaminated soils has become expensive and challenging, it is essential to prevent heavy metal pollution. Therefore, it is important to regulate the disposal of industrial wastes and sludge as well as the application of different organic soil amendments (manures, compost, bioslurry, etc.) and agro‐chemicals (chemical fertilizers and pesticides) in order to prevent heavy metals like As, Cd, Cr, Cu, Pb, Mg, Mo, Ni, Se, and Zn from contaminating the soil and crops (EPA, 2003).

Aside from the application of organic fertilizers, a large amount of chemical fertilizer has been applied worldwide to increase crop yield (Cui et al., 2018). The intensive application of chemical fertilizers has resulted in serious adverse effects on the environment, such as degradation of soil organic carbon and soil acidification (Qin et al., 2020), leaching of nutrients that cause eutrophication in aquatic and fresh water bodies (Elahi et al., 2020), and greenhouse gas emissions (Liu et al., 2020). Moreover, inorganic fertilizer availability and affordability have been a big challenge for smallholder farmers and ultimately have led to a reduction in agricultural productivity.

Beyond the use of chemical fertilizers, which has historically been a primary focus for extension agents, researchers, legislators, and donors, Ethiopia also faces a broader range of soil fertility issues. If ignored, this broader range of problems will restrict agricultural output and growth in the future throughout the nation. Chemical fertilizers may also have negative effects on the management of soil fertility and the food chain with regard to heavy metal contamination. Therefore, in order to improve soil properties, increase the balanced supply of sustainable nutrient availability, and ultimately maximize vegetable yield and food quality, it is necessary to identify and investigate a low‐cost, locally produced, and environmentally friendly source of fertilizers. Such a fertilizer could be used alone or as a supplement to chemical fertilizers. At this point, organic fertilizers derived from livestock or plants have received global support as they are the storehouse of several essential plant nutrients (Nicolodelli et al., 2016). Even though their application may increase vegetable yield and soil physicochemical characteristics, food safety and soil quality may be compromised by the use of chemical and bioslurry fertilizers. Consequently, the objective of this study was to figure out how bioslurry and chemical fertilizer influenced soil physicochemical properties, heavy metal accumulation in lettuce tissue, and the food quality of lettuce.

## 2 MATERIALS AND METHODS

### 2.1 Description of the study area

**FIGURE 1:**
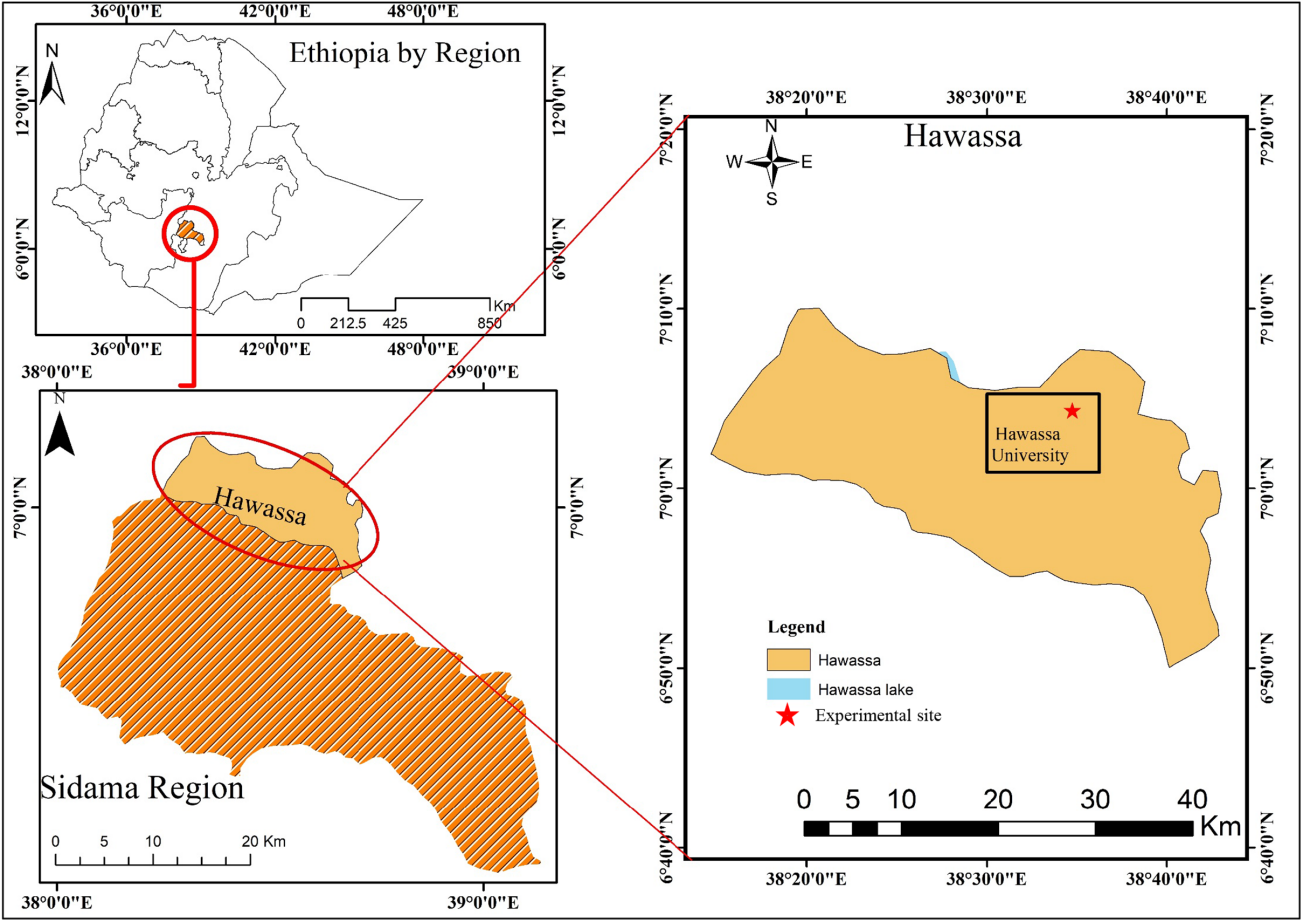
Location map of the study area.

The study was conducted at Hawassa University's Research Farm in Ethiopia (Figure 1). It is situated at 07003′53.8″ N latitude and 038028′59.2″ E longitude, 273 km south of Addis Ababa, and 1694 m above sea level.

### 2.2 Agro‐climatic condition

**FIGURE 2:**
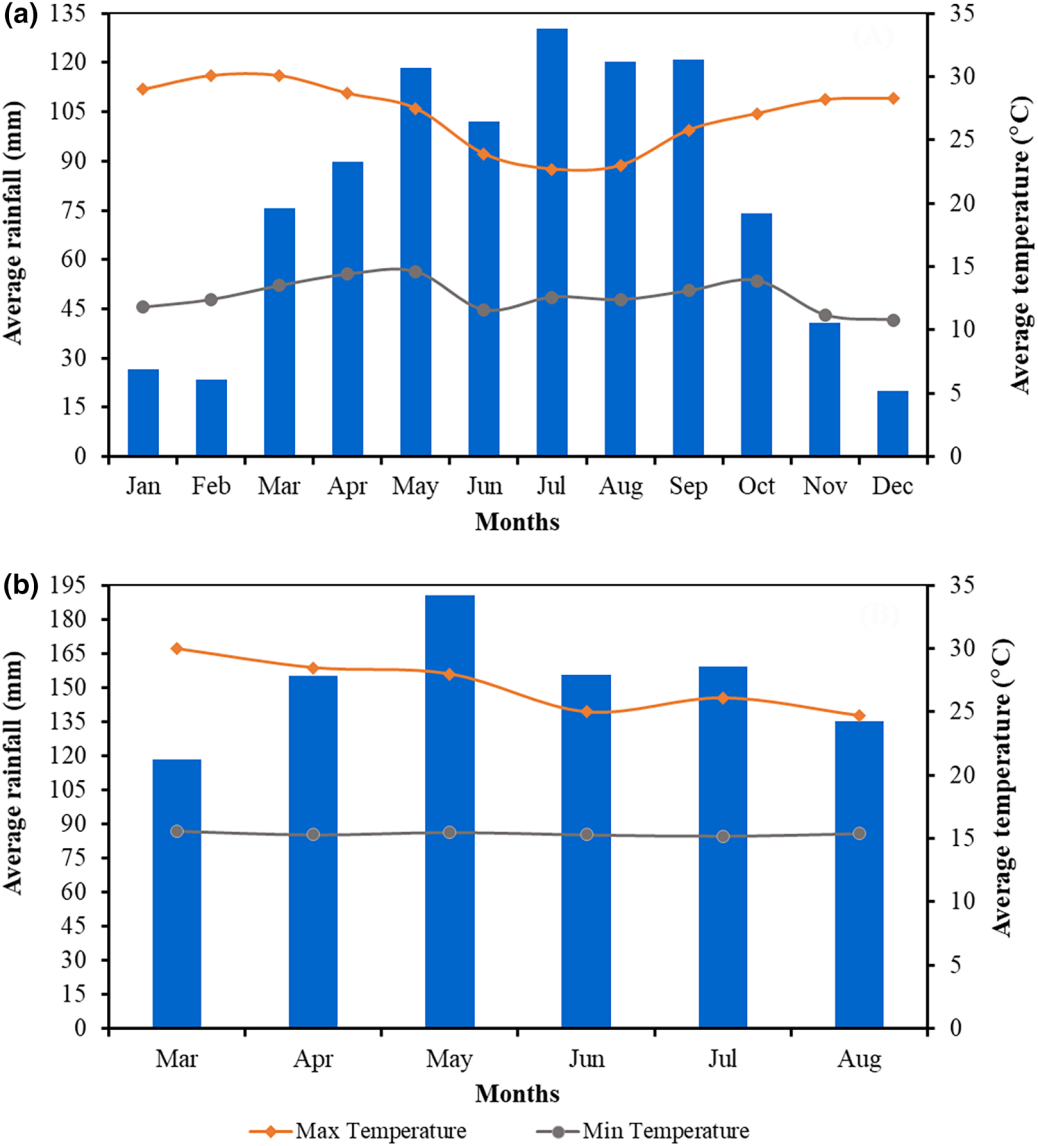
Monthly average rainfall and maximum and minimum temperature of Hawassa, recorded during 1990–2019 (a) and (b) during the experimental season (*Data source*: National Metrology Service Agency [NMSA], Hawassa Branch, 2020).

Hawassa experiences bimodal rainfall and a subhumid climate. The primary rainy season, which typically lasts from June to October, averages 996.29 mm of rainfall per year. Furthermore, the average yearly temperature is 20.9°C, with the highest temperature recorded in March (32.3°C) and the lowest in December (9.4°C) (Figure 2a). During the experimental period, the mean monthly rainfall was 146.78 mm, and the maximum and minimum temperatures were 30.8 and 14.3°C, respectively (Figure 2b).

### 2.3 Agriculture and soils

The majority of the communities in and around the study area depend heavily on agriculture. Vegetable cultivation, including lettuce, is common. The tropical Andosol soils surrounding the experimental areas have a textural class that varies from sandy loam to silty loam (Kebede et al., 2023; Lolamo et al., 2023).

### 2.4 Experimental design, treatment, and management

The experiment was laid out in a randomized complete block design with three replications. The treatments included the application of bioslurry (BS), chemical fertilizer (CF) that contained NPS and urea, and a control with no fertilizer. The plants and rows were spaced 40 and 60 cm apart, respectively (Luchia Tekle et al., 2018). Blocks and plots were spread out at 1.0 and 0.5 m, respectively. A plot measuring 3.6 × 2 m, totaling 7.2 m^2^ was used. The CF rates were 158 kg ha^−1^ NPS, 200 kg ha^−1^ urea, 5 ton ha^−1^ BS, and the control with no fertilizer. Urea was applied 4 weeks after transplanting, whereas NPS was applied at planting. The BS was incorporated into the soil at plow depth shortly before planting. *Lactuca sativa* L. cv. Parris Iceland Cos was used as a test vegetable.

**FIGURE 3:**
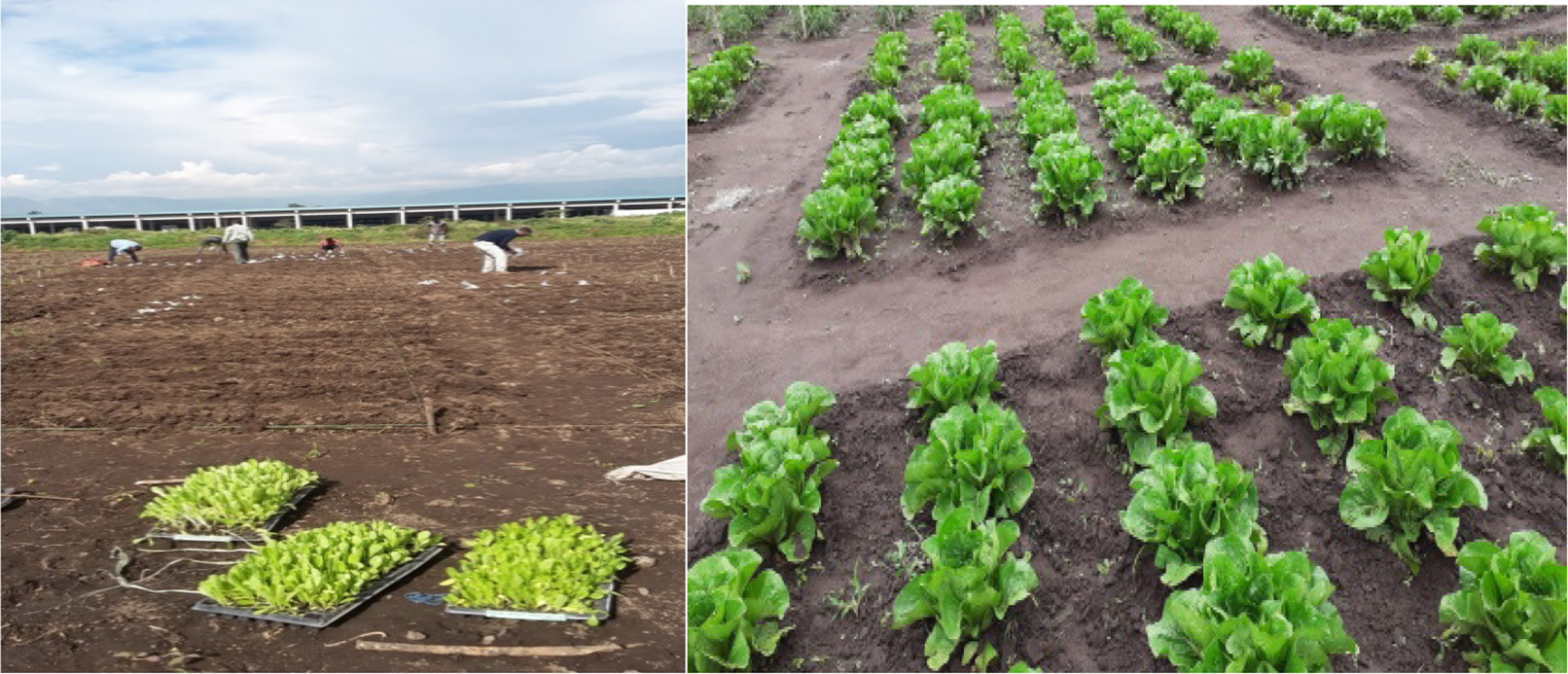
Seedlings, seedbed preparations, and experiments.

Lettuce seeds were planted in nursery trays in a net house (Figure 3) to establish lettuce seedlings. Until the final 4 days before transplanting, the trays were watered every 2 days. Six weeks after sowing, seedlings with healthy leaves were moved into the experimental plots (Figure 3). Weeding was done on a regular basis. Since none of the treatments experienced a pest infestation, no chemicals were sprayed. Harvesting was done 6 weeks after transplanting.

### 2.5 Soil, bioslurry, and lettuce sample collection, preparation, and analyses

#### 2.5.1 Soil and bioslurry sample collection and preparation

Soil (0–20 cm) samples were randomly collected from the agricultural soil (control) as well as from the agricultural soils to which BS and CF were applied. The samples were composited into one representative sample for each treatment. They were then air‐dried in the laboratory. Besides, BS samples were collected from a farmer with a biogas plant and dried in a similar way. Using pestles and a mortar, the dried soil and bioslurry samples were ground into a powder, which was then filtered through a sieve with a mesh size of 2.0 mm.

#### 2.5.2 Analysis of soil and bioslurry samples for their physico‐chemical properties

The soil and BS physico‐chemical analyses were carried out following standard methods and procedures. The major physico‐chemical properties analyzed were texture, porosity, bulk density of total nitrogen (TN), organic carbon (OC), electrical conductivity (EC), pH, cation exchange capacity (CEC), exchangeable bases (Na, Ca, and K), and available phosphorus (av. P).

#### 2.5.3 Analysis of heavy metals in soil samples

A composite soil sample (0.5 kg) from the agricultural soil was collected and added to properly labeled plastic bags. Then, the sample was transported to the HortiCoop laboratory, Bishoftu, Ethiopia, for heavy metal analysis. The soil sample was oven‐dried (25°C) for 2 days at constant weights. The dried sample was ground using a mortar and pestle, sieved with a sieve of size 2.0 mm, added to plastic bags, and stored until analysis.

In accordance, a dried 0.5 g soil sample was added into digestion vessels containing HCl, HNO_3_, and H_2_O_2_ mixtures and digested at 100°C for 2 h. Then, the digested sample was removed from the digesters, cooled down, mixed further by adding 40 mL of distilled water, and the sample was filtered using a Whatman No. 42 filter paper. Finally, the concentration of each heavy metal (As, Zn, Cd, Pb, Fe, Cu, Mn, Ni, Cr, and Co) in the digested sample was determined using Inductively Coupled Plasma Optical Emission Spectrometry (ICP‐OES: ARCOSFHS12, USA). The ICP‐OES conditions used to determine the concentrations of heavy metals are presented in Table 1.

**TABLE 1:** Instrumental operating conditions for the analysis of heavy metals in soil and lettuce leaves.

Parameters	Value
Plasma power (W)	1400
Pump speed (rpm)	30
Coolant flow (L min^−1^)	13
Auxiliary flow (L min^−1^)	0.8
Nebulizer flow (L min^−1^)	0.73
Optical temperature (°C)	14.0–16.0
Nebulizer pressure (Bar)	2.0–4.0
Main argon pressure (Bar)	6.0–8.0
Replicates	3

In addition, an individual soil sample, from each of the soils treated with BS and CF as well as from the agricultural soil, was collected and analyzed to determine the concentration level of each heavy metal, following the same procedures used for the composite soil sample above.

#### 2.5.4 Lettuce leaf sample collection and preparation for analysis

In order to analyze the levels of heavy metals in lettuce leaves, an individual fresh lettuce leaf sample, for each plot treated with BS, CF, and the control was collected. The leaf samples were cut from the lettuce plants, washed with tap water, and rinsed with de‐ionized water to remove dust particles. Then, the samples were cut into small pieces with a knife and dried in an oven at 60°C for 24 h until a constant weight was reached. The samples were then placed in labeled plastic bags and transported to the HortiCoop laboratory, Bishoftu, Ethiopia for heavy metal analysis. In the laboratory, the dried samples were crushed into powder using a mortar and pestle and then sieved with a sieve size of 2.0 mm. The sieved samples were carefully labeled, stored in polyethylene bags, and kept in desiccators until analysis.

#### 2.5.5 Analysis of lettuce leaf and bioslurry samples for heavy metal concentrations

A 0.5 g homogenized pulverized sample of each treatment was added into a digestion flask, and then 10 mL of Aqua Regia (with a 3:1 ratio of HCL to HNO_3_) and 3 mL of H_2_O_2_ were added. The mixture was heated at 300°C for 1 h on a block digester. After digestion, the mixture was filtered using Whatman No. 42 filter paper and then the filtrate was transferred to a 50‐mL volumetric flask. The samples were diluted with distilled water, and then the concentrations of heavy metals (As, Zn, Cd, Pb, Fe, Cu, Mn, Ni, Cr, and Co) were determined using ICP‐OES.

### 2.6 Statistical data analysis

Data analysis was performed using the statistical analysis system (SAS) software version 9.4 (SAS, 2011). At the 5% significance level (*p* ≤ .05), a significant difference between the treatment means was determined. When a significant difference existed, mean separation was performed using the Least Significant Difference (LSD).

Depending on the results from the laboratory analyses, the concentration level of each heavy metal in BS, soil treated with BS and CF, and lettuce leaf was determined and compared with the maximum permissible limit set for agricultural soils and crops (FAO/WHO, 2011).

## 3 RESULTS AND DISCUSSION

### 3.1 Physico‐chemical properties of the agricultural soil and bioslurry

The agricultural soil had a loam texture, a porosity of 62.9%, and a bulk density of 1 g cm^−3^. The soil was moderately acidic in reaction, with a pH value of 5.64, ranging between 5.6 and 6.0 (Rowell, 2014). The OC (2.73%) is in a medium range (Landon, 1991), and so is the TN (0.24%) (Tadese, 1991). The available P (49.6 ppm) is classified as high range (Olsen et al., 1954). The agricultural soil used for this study had long been used for experiments on various agricultural research projects employing larger amounts of chemical fertilizers and pesticides, which could be the most likely cause of the increased N and P in the agricultural soil. Findings from previous studies also concur with the present finding: long term and overapplication of chemical fertilizers or pesticides could increase soil N and P and other soil phyico‐chemical properties (Baweja et al., 2020; Cui et al., 2020; Pahalvi et al., 2021). The concentrations of Ca (46.15 cmol kg^−1^), Na (0.21 cmol kg^−1^), and K (2.29 cmol kg^−1^) are, respectively, high, low, and very high (Hazelton & Murphy, 2016). The CEC (24.6 cmol kg^−1^) is in the medium range (Landon, 1991).

The bioslurry had a pH of 7.52, OC of 6.24%, TN of 0.54%, C:N of 11.56, available P of 262.2 ppm, and CEC of 39 cmol kg^−1^, as well as exchangeable K, Ca, and Na of 10.3, 52.34, and 0.39 cmol kg^−1^, respectively. This result is consistent with previous studies showing that bioslurry has similar phyico‐chemical characteristics (Kinaichu et al., 2021; Nuhriawangsa et al., 2021).

### 3.2 Heavy metal concentrations in agricultural soil and bioslurry

The mean concentrations of As, Pb, Zn, Cd, Cu, Ni, Co, Fe, Mn, and Cr in the agricultural soil were found to be 16.60, 28.17, 11.23, 4.34, 0.54, 3.30, 0.33, 213.13, 129.86, and 4.54 mg kg^−1^, respectively. The maximum permissible limits (MPLs) of the same heavy metals for agricultural soil are 20, 50, 1000, 3, 300, 50, 50, 50,000, 2000, and 75 mg kg^−1^, respectively (Table 2). Moreover, the concentrations of As, Pb, Zn, Cd, Cu, Ni, Co, Fe, Mn, and Cr in the bioslurry were 10.48, 17.69, 66.13, 8.80, 7.57, 3.59, 13.39, 550.87, 322.90, and 0.26 mg kg^−1^, respectively.

**TABLE 2:** Mean concentration (mean ± SD) of heavy metals in control soil and bioslurry.

Sample	Heavy metal concentration (mg kg^−1^ dry weight)									
	As	Pb	Zn	Cd	Cu	Ni	Co	Fe	Mn	Cr
Soil	16.60 ± 2.76	28.17 ± 1.20	11.23 ± 0.93	4.34 ± 0.90	0.54 ± 0.002	3.30 ± 0.32	0.33 ± 0.01	213.13 ± 6.56	129.86 ± 4.47	4.54 ± 0.12
Bioslurry	10.48 ± 0.63	17.69 ± 0.83	66.13 ± 3.1	8.80 ± 0.22	7.57 ± 0.79	3.59 ± 0.54	13.39 ± 0.33	550.87 ± 15.20	322.90 ± 4.94	0.26 ± 0.03
MPL	20.0	50.0	1000.0	3.0	300.0	50.0	50	50,000	2000	75.0

Abbreviations: MPL, maximum permissible limit for agricultural soils according to FAO/WHO (2011); SD, standard deviation.

The mean concentration of Cd in the soil was 4.34 mg kg^−1^ while its MPL for the agricultural soil is 3 mg kg^−1^ (FAO/WHO, 2011). Thus, the concentration of Cd in the soil was above the MPL of FAO/WHO (2011) for agricultural soil. However, the mean concentrations of the other heavy metals in the soil were below the MPL of FAO/WHO (2011) for agricultural soil (Table 2). This demonstrates that the soil is slightly contaminated with Cd.

### 3.3 Application of chemical fertilizer and bioslurry and soil physicochemical characteristics

The application of bioslurry increased porosity, whereas it decreased bulk density. It also improved soil pH, TN, OC, available P, CEC, and exchangeable K and Na (Table 3). These results comply with previous findings that organic fertilizers improve soil fertility and aggregates (Sayara et al., 2020), improve soil structure, increase porosity, and decrease bulk density, which provides a healthy soil environment (Bassouny & Abuzaid, 2017). Likewise, the application of chemical fertilizer slightly increased soil porosity and decreased bulk density, although this effect was minimal compared to that of bioslurry. Apart from that, unlike bioslurry, the application of chemical fertilizer slightly reduced OC, TN, OM, pH, exchangeable bases, and remarkably reduced the quantity of available P (Table 3). This shows that bioslurry and chemical fertilizer have dissimilar effects on some of the physicochemical characteristics of the soil. These results are consistent with findings from previous studies (Kebede et al., 2023; Lolamo et al., 2023; Nasir et al., 2012).

**TABLE 3:** Effects of bioslurry and chemical fertilizer on soil physicochemical properties.

Soil physicochemical properties	Control	Bioslurry	Chemical fertilizer
Bulk density (BD, g cm^−3^)	0.98	0.93	0.99
Porosity (%)	62.9	64.8	62.5
Texture	Sandy Clay Loam	Sandy Clay Loam	Sandy Clay Loam
Organic carbon (OC, %)	2.34	2.73	2.15
Total nitrogen (TN, %)	0.204	0.238	0.187
Organic matter (OM, %)	4.08	4.76	3.74
Available Phosphorus (P, ppm)	70.2	72.84	39.36
pH	5.37	5.92	5.16
Electrical conductivity (EC, ms m^−1^)	1.07	0.98	2.11
Catio exchange capacity (CEC, cmol kg^−1^)	23.8	24.4	24.4
Exchangeable sodium (cmol kg^−1^)	0.22	0.23	0.19
Exchangeable calcium (cmol kg^−1^)	58.6	50.62	46.39
Exchangeable potassium (cmol kg^−1^)	2.44	2.53	2.06

Bioslurry increased soil pH, while chemical fertilizers reduced it. The application of bioslurry increased soil pH by 4.73% and 14.73% compared to the agricultural soil and soil treated with chemical fertilizer, respectively. The increased pH attributes to the near‐neutral or slightly alkaline pH of the bioslurry (Table 3). Aside from that, the application of bioslurry offered higher OC, OM, and TN compared to the chemical fertilizers (Table 3). In line with the current results, Adal et al. (2024) reported an increase in soil pH and OC upon amendment with bioslurry. Furthermore, the application of bioslurry increased CEC, available P, Na, and K compared with the control (Table 3), indicating its higher residual effects in improving soil characteristics. Organic fertilizers have the advantages of building up soil organic matter and enhancing soil health (Nasir et al., 2012; Shahbaz et al., 2014), increasing soil fertility, improving nutrient retention, and preventing cations from leaching (Adugna, 2016). Apart from that, bioslurry increased exchangeable base and CEC (Mofokeng et al., 2020) and increased OC and P in the soil (Debebe & Itana, 2016).

### 3.4 Heavy metal concentration in agricultural soil

Table 4 presents the mean heavy metal concentrations in soil samples of all treatments. Low levels of some metals, such as Fe, Zn, Mn, Ni, and Cu, are important for plants and animals, while many other metals, such as Cr, Cd, Pb, Co, and As, are not (Alloway, 2013). Organic fertilizers are more effective in reducing Cr, Cd, Mn, and Pb uptake, improving plant growth and food quality and reducing human‐health risk (Alam et al., 2020).

**TABLE 4:** The effects of bioslurry and chemical fertilizer application on the mean concentrations of heavy metals (mg kg^−1^) compared to MPL.

Heavy metals	Heavy metal concentration (mg kg^−1^)			Threshold
	Control soil	Bioslurry‐treated soil	Chemical fertilizer‐treated soil	MPL
Arsenic	16.6 ± 2.757	15.53 ± 0.439	13.95 ± 1.355	20
Lead	28.2 ± 1.200	21.7 ± 0.750	23.48 ± 0.817	50
Zinc	11.2 ± 0.934	12.7 ± 0.934	11.3 ± 0.133	1000
Cadmium	4.34 ± 0.896	3.89 ± 0.468	3.78 ± 0.579	3
Copper	0.54 ± 0.003	0.85 ± 0.037	0.6 ± 0.046	300
Nickel	3.3 ± 0.316	2.5 ± 0.270	2.24 ± 0.064	50
Cobalt	0.33 ± 0.014	0.29 ± 0.034	0.27 ± 0.031	50
Iron	213 ± 6.556	110.5 ± 7.040	120.9 ± 3.822	1500
Manganese	129.9 ± 4.473	131.4 ± 4.394	120.7 ± 4.033	600
Chromium	4.54 ± 0.121	3.95 ± 0.230	3.17 ± 0.143	75

Abbreviation: MPL, maximum permissible limit for agricultural soil according WHO/FAO (2011).

#### 3.4.1 Cadmium

Across the three soil samples, the mean concentration of Cd surpassed the MPL set by WHO/FAO (2011) for agricultural soils, whereas the concentrations of other heavy metals were below the MPL. The highest mean concentration of Cd was obtained from the agricultural soil, while the smallest concentration was recorded in the chemical fertilizer‐treated soil (Table 4). The Cd concentration in agricultural soil was higher by 11.57% and 14.82% compared to the soils treated with bioslurry and chemical fertilizer, respectively. The agricultural soil has long been used for conducting agricultural experiments, which might be the most likely source of the higher Cd concentration, which concurs with a previous study uncovering that the residual effects of various kinds of agro‐chemicals, including pesticides, could result in increased heavy metal concentrations (Amjad et al., 2017). There are more concurring studies, including that the increased concentration level in the agricultural soil could be attributed to the naturally occurring Cd in soils with lower pH (Ubuoh et al., 2019), and a significantly higher Cd concentration (2–4 mg kg^−1^) was reported in agricultural soil in Peru (Oliva et al., 2019). Apart from that, the Cd concentration in soil treated with bioslurry was greater than that in soil treated with chemical fertilizer, which could be attributed to higher concentration of Cd in bioslurry and agricultural soil (Table 2). This result is consistent with a finding from a previous study that bioslurry and organic manure application can result in the concentration of heavy metals in soils (Pirsaheb et al., 2016). The additional probable potential sources of Cd in the agricultural soils of the present study could also be its proximity to chemical industries.

#### 3.4.2 Lead

Across all treatments, the concentration of Pb did not exceed the MPL set by WHO/FAO (2011) for agricultural soils. Compared to the soil treated with chemical fertilizer and bioslurry, the highest Pb concentration was recorded in the agricultural soil (Table 4). Especially, the lower pH of the agricultural soil and contaminants from various sources, including natural or anthropogenic, might have contributed to the increased availability of Pb. For a similar reason, the chemical fertilizer‐treated soil had greater concentration of Pb than the bioslurry‐treated soil (Table 4). This might be because the application of chemical fertilizer lowered the pH (Table 3). Lower soil pH increases the bioavailability of Pb (Zhang et al., 2018). Chemical fertilizer application increased the concentration of Pb in soils (Atafar et al., 2010). Moreover, the lower concentration of Pb due to bioslurry application is consistent with a previous study that found organic fertilizer application could reduce Pb and other heavy metal concentrations in soils (Lin et al., 2019).

#### 3.4.3 Arsenic

The concentration of As in all soil samples did not exceed the MPL set by the WHO/FAO (2011) for agricultural soils. The soil treated with chemical fertilizers had the lowest As concentration, while agricultural soil had the highest concentration. The highest concentration in the agricultural soil could have stemmed from its lower pH (Table 3) and other potential natural and anthropogenic sources. On top of that, the concentration of As in the soil treated with bioslurry was greater than that in the soil treated with chemical fertilizers, which attributes to the existence of higher As in the bioslurry and in the study soil (Table 2). Organic amendments could lead to the accumulation of As in the soil (Wuana & Okieimen, 2011), and natural and anthropogenic activities could add As to the soil (Bencko et al., 2017).

#### 3.4.4 Cobalt

The mean concentration of Co was under the threshold set by the WHO/FAO (2011) for agricultural soil (Table 4). The concentration of Co in the soil treated with bioslurry exceeded that of the soil treated with chemical fertilizers. This might be attributed to the fact that the applied bioslurry had a higher concentration of Co (Table 2) and the control soil had a lower pH and a higher concentration of Co (Table 3), which make favorable condition for the higher concentration of Co in the soil treated with bioslurry (Table 4). The current result is in line with earlier research findings (Laghrib et al., 2021; Shen et al., 2020).

#### 3.4.5 Chromium

The mean concentration of Cr was under the threshold set by FAO/WHO (2011) for agricultural soils (Table 4). The soil treated with chemical fertilizers had the lowest concentration, while agricultural soil had the highest concentration. The soil treated with bioslurry had a higher concentration of Cr than the soil treated with chemical fertilizer, which appears to result from the higher concentration of Cr in the bioslurry (Table 2). This finding also supports the notion that adding organic fertilizers to soils increases the bioavailability of Cr (Sinduja et al., 2023).

#### 3.4.6 Zinc, copper, manganese, nickel, and iron

The agricultural soil had the highest concentrations of Ni and Fe, whereas the soil treated with chemical fertilizers and bioslurry had the lowest concentrations (Table 2). However, the greatest concentration of Co, Mn, and Zn was recorded in the bioslurry‐treated soil, while the lowest was in the chemical fertilizer‐treated soil (Table 4). The increased concentration of Co, Mn, and Zn in bioslurry‐treated soil might stem from the existence of Cu, Mn, and Zn in bioslurry and agricultural soil (Table 2). Moreover, compared with chemical fertilizers, organic fertilizers often contain a greater level of heavy metals, which could enter the soil through application (Lolamo et al., 2023). Organic fertilizer amendments could result in more accumulation of Zn in soils than chemical fertilizers (Ning et al., 2017).

In general, with the exception of Cd, the levels of concentrations of heavy metals in soils sampled from all treatments were below the threshold for agricultural soil (FAO/WHO, 2011). This demonstrates that, for the majority of the heavy metals under study, the soils from all treatments may be suitable for lettuce cultivation. This needs to be carefully interpreted, though, as vegetables grown in these soils may absorb heavy metals, which may then accumulate and be passed on to other living things via the food chain.

### 3.5 The concentration of heavy metals in lettuce

Table 5 presents the mean concentrations of heavy metals in lettuce samples. These concentrations were sequenced as Fe > Mn > Zn > Ni > As > Cr for the agricultural soil, Fe > Mn > Zn > Ni > Cr for the chemical fertilizer‐treated soil and Fe > Mn > Zn > Pb > Ni > As > Co > Cd > Cr for the bioslurry‐treated soil. Heavy metal contamination of soil is a frequent occurrence and can be a significant source of metals for crops, which may eventually enter the food chain and expose people to these potentially harmful metals (Ahmad et al., 2019).

**TABLE 5:** The concentrations of heavy metals in lettuce for the different treatments (mg kg^−1^).

Heavy metals	Concentration of heavy metals (mg kg^−1^) threshold			
	Control soil	Bioslurry‐treated soil	Chemical fertilizer‐treated soil	MPL
Arsenic	5.1 ± 0.414	11.2 ± 0.091	ND	0.03
Lead	ND	16.7 ± 0.580	ND	0.3
Zinc	23.5 ± 0.386	24 ± 1.757	26 ± 0.816	99.4
Cadmium	ND	7.0 ± 1.050	ND	0.02
Copper	ND	ND	ND	73.3
Nickel	10.9 ± 0.281	12.1 ± 0.893	14.44 ± 1.381	66.9
Cobalt	ND	7.3 ± 0.771	ND	50
Iron	312.2 ± 16.012	318 ± 5.052	218.5 ± 5.209	425.5
Manganese	53 ± 3.643	44.6 ± 4.490	83.4 ± 4.736	500
Chromium	0.202 ± 0.003	0.24 ± 0.020	0.12 ± 0.022	20

Abbreviations: MPL, maximum permissible limit for vegetables according to WHO/FAO, 2011; ND, below detection limit.

The concentrations of heavy metals in the lettuce, from different treatments, were found to be varied. The concentration of As (5.1 mg kg^−1^) in the lettuce sample from the agricultural soil and As (11.2 mg kg^−1^), Pb (16.7 mg kg^−1^), and Cd (7.0 mg kg^−1^) in the lettuce sample grown in the bioslurry‐treated soils exceeded the heavy metal threshold level set by WHO/FAO (2011) for vegetables, whereas the concentration of Zn, Cu, Ni, Co, Fe, Mn, and Cr in the lettuce sample collected from different treatments were below the MPL (Table 5).

#### 3.5.1 Cadmium concentration

The lettuce sample that was grown in soil treated with bioslurry had the highest concentration of Cd (Table 5), while it was below the detection limit in agricultural soil and chemical fertilizer‐treated soil (Table 2). The Cd in the lettuce sample from the bioslurry‐treated soil might stem from the Cd contained in the bioslurry and control soil and other potential inherent sources (Table 2). Soil organic fertilizer amendments could increase the bioavailability of Cd in soil and ultimately its uptake by plants (Rehman et al., 2020). Heavy metal cations adsorb organic matter in the soil and are taken up by plants (Wang et al., 2018). These conditions might be the reason for the concentration of Cd in the bioslurry‐grown lettuce exceeding the MPL set by WHO/FAO (2011). This proves that the toxic levels of Cd in lettuce make it unsafe for human consumption. Excessive consumption of foods with high Cd concentrations has been linked to severe vomiting, diarrhea, cancer, bone fractures, and damage to the central nervous system (Alengebawy et al., 2021; Tegegne, 2015).

#### 3.5.2 Lead concentration

The highest concentration of Pb was recorded in lettuce grown in the bioslurry‐treated soil, which surpassed the MPL set by the WHO/FAO (2011). Nonetheless, Pb was not detected in the remaining treatments (Table 5). The high Pb in bioslurry‐grown lettuce might have resulted from the higher Pb concentration in bioslurry and bioslurry‐treated soil (Table 2), the lower pH of the agricultural soil (Table 3), affinities of lettuce for uptake of Pb from the soil, and other soil‐inherent sources. Therefore, the consumption of the high concentration of Pb in lettuce grown with bioslurry treatment may pose a human health risk. This is due to the fact that lead poisoning raises the risk of oxidative stress, changes in sodium ion concentration, neurological disorders, other serious health issues, and even death (Amaravadi et al., 2019; Khanam et al., 2020).

#### 3.5.3 Arsenic concentration

The highest concentration of As occurred in bioslurry‐treated lettuce, while the lowest occurred in chemical fertilizer‐treated lettuce (Table 5). The concentration of As in bioslurry‐treated soil and agricultural soil exceeded the maximum permissible limit of heavy metals in vegetables (WHO/FAO, 2011). The highest As concentration in the bioslurry‐treated lettuce might stem from the higher heavy metal concentration in the agricultural soil and bioslurry (Table 2) and other potential factors. On top of that, the high concentration of As in the lettuce grown in the agricultural soil might be attributed to its high concentration and its lower pH. Organic fertilizer applications, including livestock manure and slurries, greatly increase the amount of nutrients available to crops through the absorption of heavy metals (Wang et al., 2018). As a whole, the lettuce cultivated in the soil treated with bioslurry and in agricultural soil is toxic to humans. Exposure to As may lead to risks of skin lesions, such as hyperkeratosis and pigmentation changes, as well as skin cancer (Mandal, 2017; Rahaman et al., 2021).

#### 3.5.4 Concentration of zinc, copper, nickel, cobalt, iron, manganese, and chromium

The highest concentrations of Co, Fe, and Cr occurred in the bioslurry lettuce, while the lowest occurred in the chemical fertilizer lettuce (Table 5). Nevertheless, none of these concentrations exceeded the maximum permissible limit recommended by the WHO/FAO (2011) for vegetables. Aside from that, the level of concentration of Mn, Ni, and Zn was the highest in the lettuce treated with chemical fertilizer, while it was the lowest in the lettuce grown in agricultural soil. Nevertheless, none of these concentrations exceeded the maximum permissible limit (WHO/FAO, 2011) for vegetables (Table 5). The existence of heavy metals in lettuce might stem from the higher concentrations of metals in bioslurry and the agricultural soil (Table 1), which for a long time had been used for agricultural research. Long‐term applications of P fertilizers and pesticides are likely sources of heavy metals in agricultural soils and crops (AlKhader, 2015).

In general, the concentrations of Pb, Cd, and As in lettuce grown in bioslurry‐treated soil and As in agricultural soil exceeded the recommended limit (WHO/FAO, 2011) for vegetables (Table 5). This indicates that lettuce grown with the application of bioslurry is not safe for human consumption regarding As, Pb, and Cd toxicities in the study sites. Likewise, lettuce grown in control soil was unsafe for human consumption due to its As toxicities. The elevated concentrations of heavy metals in the bioslurry, which in turn raised the heavy metal concentrations in the soil and lettuce, may have been caused by the feedstock's high heavy metal concentrations (Table 2). Yet, the rest of the heavy metals, such as Zn, Co, Fe, Mn, Ni, and Cr, fell under the safe limit recommended by WHO/FAO (2011) for vegetables. Also, the lettuce grown with chemical fertilizer treatment was safe for human consumption for all heavy metals, as they were all found under the threshold limit set by WHO/FAO (2011) for vegetables.

## 4 CONCLUSIONS AND RECOMMENDATIONS

While the physico‐chemical characteristics of the soil were improved by applying bio‐slurry, some of the most important heavy metals were increased in both the soil and the lettuce. The application of bio‐slurry increased the concentrations of Cd, As, and Pb in lettuce samples, which surpassed the upper limit for vegetables suggested by WHO/FAO (2011). Because of the toxicities of As, Pb, and Cd, consuming lettuce cultivated in bio‐slurry‐amended soils may be unsafe for human health. The most likely sources of these heavy metals might be the nature of the feedstock for bio‐slurry preparation. The higher level concentration of these metals in the agricultural soil are also the most likely sources of the heavy metals in lettuce. As this study was conducted only for one cropping season and with bio‐slurry sourced from a specific feedstock, more research is necessary to evaluate the potential impacts of bio‐slurry and chemical fertilizer application on soil physicochemical characteristics, heavy metal concentrations, and the food safety of vegetables, taking into account bio‐slurry sourced from different feedstocks, application rates, and soil types under field conditions. At some point, we advise that when growing vegetables, the physico‐chemical characteristics of agricultural soils and bio‐slurry be taken into account for their heavy metal concentrations and potential toxicities.

## AUTHOR CONTRIBUTIONS

**Tsigereda Meskelu:** Formal analysis (equal); investigation (equal); methodology (equal); software (equal); validation (equal); visualization (equal); writing – original draft (equal); writing – review and editing (equal). **Yadessa Gonfa Keneni:** Conceptualization (supporting); data curation (equal); formal analysis (equal); funding acquisition (equal); investigation (equal); methodology (equal); project administration (supporting); resources (equal); software (equal); supervision (lead); validation (equal); visualization (equal); writing – original draft (equal); writing – review and editing (equal). **Abate Feyissa Senbeta:** Conceptualization (lead); data curation (equal); formal analysis (equal); funding acquisition (equal); investigation (equal); methodology (equal); project administration (equal); resources (equal); software (equal); supervision (lead); validation (equal); visualization (equal); writing – original draft (equal); writing – review and editing (equal). **Getachew Sime:** Conceptualization (equal); data curation (equal); formal analysis (equal); funding acquisition (lead); investigation (equal); methodology (equal); project administration (lead); resources (lead); software (equal); supervision (equal); validation (equal); visualization (equal); writing – original draft (equal); writing – review and editing (equal).

## FUNDING INFORMATION

This study was funded by Hawassa University, Ethiopia.

## CONFLICT OF INTEREST STATEMENT

Authors declare no conflicts of interests.

## Data Availability

The manuscript itself contains the data that were used in the present study.

## References

[fsn34363-bib-0001] Adal, M. , Ali, Y. , Wubie, G. , & Muluneh, G. (2024). Effect of liquid bio‐slurry and chemical fertilizer applications on growth and yield response of potato (*Solanum tuberosum*) and soil properties. International Journal of Agriculture and Biology, 31(1), 65–76.

[fsn34363-bib-0002] Adugna, G. (2016). A review on impact of compost on soil properties, water use and crop productivity. Academic Research Journal of Agricultural Science and Research, 4(3), 93–104.

[fsn34363-bib-0003] Ahmad, K. , Wajid, K. , Khan, Z. I. , Ugulu, I. , Memoona, H. , Sana, M. , Nawaz, K. , Malik, I. S. , Bashir, H. , & Sher, M. (2019). Evaluation of potential toxic metals accumulation in wheat irrigated with wastewater. Bulletin of Environmental Contamination and Toxicology, 102, 822–828. 30955046 10.1007/s00128-019-02605-1

[fsn34363-bib-0004] Alam, M. , Hussain, Z. , Khan, A. , Khan, M. A. , Rab, A. , Asif, M. , Shah, M. A. , & Muhammad, A. (2020). The effects of organic amendments on heavy metals bioavailability in mine impacted soil and associated human health risk. Scientia Horticulturae, 262, 109067.

[fsn34363-bib-0005] Alberdi, H. A. , Sagala, S. A. , Wulandari, Y. , Srajar, S. L. , & Nugraha, D. (2018). Biogas implementation as waste management effort in Lembang sub‐district, West Bandung District. In IOP Conference Series: Earth and environmental science (Vol. 158(1), 012031). IOP Publishing.

[fsn34363-bib-0006] Alengebawy, A. , Abdelkhalek, S. T. , Qureshi, S. R. , & Wang, M. Q. (2021). Heavy metals and pesticides toxicity in agricultural soil and plants: Ecological risks and human health implications. Toxics, 9(3), 42. 33668829 10.3390/toxics9030042 PMC7996329

[fsn34363-bib-0007] AlKhader, A. M. F. (2015). The impact of phosphorus fertilizers on heavy metals content of soils and vegetables grown on selected farms in Jordan. Agrotechnology, 5(1), 137.

[fsn34363-bib-0008] Alloway, B. J. (2013). Heavy metals and metalloids as micronutrients for plants and animals. In B. J. Alloway (Ed.), Heavy metals in soils: Trace metals and metalloids in soils and their bioavailability (pp. 195–209). Springer Netherlands.

[fsn34363-bib-0009] Amaravadi, R. K. , Kimmelman, A. C. , & Debnath, J. (2019). Targeting autophagy in cancer: Recent advances and future directions targeting autophagy in cancer. Cancer Discovery, 9(9), 1167–1181. 31434711 10.1158/2159-8290.CD-19-0292 PMC7306856

[fsn34363-bib-0010] Amjad, A. L. I. , Di, G. U. O. , Mahar, A. , Ping, W. A. N. G. , Feng, S. H. E. N. , Ronghua, L. I. , & Zhang, Z. (2017). Mycoremediation of potentially toxic trace elements—A biological tool for soil clean up: A review. Pedosphere, 27(2), 205–222.

[fsn34363-bib-0011] Atafar, Z. , Mesdaghinia, A. , Nouri, J. , Homaee, M. , Yunesian, M. , Ahmadimoghaddam, M. , & Mahvi, A. H. (2010). Effect of fertilizer application on soil heavy metal concentration. Environmental Monitoring and Assessment, 160, 83–89. 19058018 10.1007/s10661-008-0659-x

[fsn34363-bib-0012] Bassouny, M. A. , & Abuzaid, A. S. (2017). Impact of biogas slurry on some physical properties in sandy and calcareous soils, Egypt. International Journal of Plant & Soil Science, 16, 1–11.

[fsn34363-bib-0013] Baweja, P. , Kumar, S. , & Kumar, G. (2020). Fertilizers and pesticides: Their impact on soil health and environment. In B. Giri & A. Varma (Eds.), Soil health. Soil biology (pp. 265–285). Springer.

[fsn34363-bib-0014] Bencko, V. , & Foong, Y. L. F. (2017). The history of arsenical pesticides and health risks related to the use of Agent Blue. Annals of Agricultural and Environmental Medicine, 24(2), 312–316. 28664715 10.26444/aaem/74715

[fsn34363-bib-0015] Bhardwaj, P. , Sharma, R. K. , Chauhan, A. , Ranjan, A. , Rajput, V. D. , Minkina, T. , & Tripathi, A. (2023). Assessment of heavy metal distribution and health risk of vegetable crops grown on soils amended with municipal solid waste compost for sustainable urban agriculture. Water, 15(2), 228.

[fsn34363-bib-0016] Bigdeli, M. , & Seilsepour, M. (2008). Investigation of metals accumulation in some vegetables irrigated with waste water in Shahre Rey‐Iran and toxicological implications. American‐Eurasian Journal of Agricultural & Environmental Sciences, 4(1), 86–92.

[fsn34363-bib-0017] Cui, N. , Cai, M. , Zhang, X. , Abdelhafez, A. A. , Zhou, L. , Sun, H. , Chen, G. , Zou, G. , & Zhou, S. (2020). Runoff loss of nitrogen and phosphorus from a rice paddy field in the east of China: Effects of long‐term chemical N fertilizer and organic manure applications. Global Ecology and Conservation, 22, e01011.

[fsn34363-bib-0018] Cui, Z. , Zhang, H. , Chen, X. , Zhang, C. , Ma, W. , Huang, C. , Zhang, W. , Mi, G. , Miao, Y. , Li, X. , Gao, Q. , Yang, J. , Wang, Z. , Ye, Y. , Guo, S. , Lu, J. , Huang, J. , Lv, S. , Sun, Y. , … Dou, Z. (2018). Pursuing sustainable productivity with millions of smallholder farmers. Nature, 555(7696), 363–366. 29513654 10.1038/nature25785

[fsn34363-bib-0019] Debebe, Y. , & Itana, F. (2016). Comparative study on the effect of applying biogas slurry and inorganic fertilizer on soil properties, growth, and yield of white cabbage (Brassica oleracea var. capitata f. alba). Journal of Biology, Agriculture and Healthcare, 6(19), 19–26.

[fsn34363-bib-0020] Elahi, A. , Arooj, I. , Bukhari, D. A. , & Rehman, A. (2020). Successive use of microorganisms to remove chromium from wastewater. Applied Microbiology and Biotechnology, 104, 3729–3743. 32172324 10.1007/s00253-020-10533-y PMC13521037

[fsn34363-bib-0021] EPA . (2003). Environmental Protection Authority (EPA).

[fsn34363-bib-0022] Faran, M. , Nadeem, M. , Manful, C. F. , Galagedara, L. , Thomas, R. H. , & Cheema, M. (2023). Agronomic performance and phytochemical profile of lettuce grown in anaerobic dairy digestate. Agronomy, 13(1), 182.

[fsn34363-bib-0023] Food and Agricultural Organization (FAO)/World health organization (WHO) . (2011). Joint FAO/WHO food standards programme—Codex Alimentarius Commission . Report of the 34th session of the codex committee on food additives and contaminants. Switzerland.

[fsn34363-bib-0024] Gebeyehu, H. R. , & Bayissa, L. D. (2020). Levels of heavy metals in soil and vegetables and associated health risks in Mojo area, Ethiopia. PLoS One, 15(1), e0227883. 31999756 10.1371/journal.pone.0227883 PMC6992214

[fsn34363-bib-0025] Hazelton, P. , & Murphy, B. (2016). Interpreting soil test results: What do all the numbers mean (2nd ed., p. 152). CSIRO Publishing.

[fsn34363-bib-0026] Kebede, T. , Keneni, Y. G. , Senbeta, A. F. , & Sime, G. (2023). Effect of bioslurry and chemical fertilizer on the agronomic performances of maize. Heliyon, 9, e13000. 36711291 10.1016/j.heliyon.2023.e13000 PMC9873682

[fsn34363-bib-0027] Khan, A. , Khan, S. , Alam, M. , Khan, M. A. , Aamir, M. , Qamar, Z. , Ur Rehman, Z. , & Perveen, S. (2016). Toxic metal interactions affect the bioaccumulation and dietary intake of macro‐and micro‐nutrients. Chemosphere, 146, 121–128. 26714294 10.1016/j.chemosphere.2015.12.014

[fsn34363-bib-0028] Khanam, R. , Kumar, A. , Nayak, A. K. , Shahid, M. , Tripathi, R. , Vijayakumar, S. , Bhaduri, D. , Kumar, U. , Mohanty, S. , Panneerselvam, P. , Chatterjee, D. , Satapathy, B. S. , & Pathak, H. (2020). Metal (loid) s (As, Hg, Se, Pb and Cd) in paddy soil: Bioavailability and potential risk to human health. Science of the Total Environment, 699, 134330. 31522043 10.1016/j.scitotenv.2019.134330

[fsn34363-bib-0029] Kinaichu, J. G. , Nyaga, C. G. , Njogu, P. , Gatebe, E. G. , & Maina, E. G. (2021). Comparative study of selected macro and micronutrients in bio slurry samples from different feed stocks and inorganic fertilizers. Asian Journal of Applied Chemistry Research, 2, 1–7.

[fsn34363-bib-0030] Laghrib, F. , Saqrane, S. , Lahrich, S. , & El Mhammedi, M. A. (2021). Best of advanced remediation process: Treatment of heavy metals in water using phosphate materials. International Journal of Environmental Analytical Chemistry, 101(9), 1192–1208.

[fsn34363-bib-0031] Landon, J. R. (1991). Booker tropical soil manual: A handbook for soil survey and agricultural land evaluation in the tropics and subtropics (p. 474). Long man Scientific and Technical Essex.

[fsn34363-bib-0032] Lin, W. , Lin, M. , Zhou, H. , Wu, H. , Li, Z. , & Lin, W. (2019). The effects of chemical and organic fertilizer usage on rhizosphere soil in tea orchards. PLoS One, 14(5), e0217018. 31136614 10.1371/journal.pone.0217018 PMC6538140

[fsn34363-bib-0033] Liu, X. , Elgowainy, A. , & Wang, M. (2020). Life cycle energy use and greenhouse gas emissions of ammonia production from renewable resources and industrial by‐products. Green Chemistry, 22(17), 5751–5761.

[fsn34363-bib-0034] Liu, Y. , Ma, R. , Yang, Y. , Wang, J. , Guan, X. , Wang, M. , & Jiang, L. (2023). Effect of partial organic fertilizer substitution on heavy metal accumulation in wheat grains and associated health risks. Agronomy, 13(12), 2930.

[fsn34363-bib-0035] Lolamo, T. , Senbeta, A. F. , Keneni, Y. G. , & Sime, G. (2023). Effects of bio‐slurry and chemical fertilizer application on soil properties and food safety of tomato (*Solanum lycopersicum* Mill.). Applied and Environmental Soil Science, 2023(1), 6694536.

[fsn34363-bib-0036] Madar, Á. K. , & Hájos, M. T. (2021). Evolution of quality parameters of different lettuce (*Lactuca sativa* L.) varieties under unheated plastic tunnel. Agriculture and Environment, 13, 88–99.

[fsn34363-bib-0037] Mandal, P. (2017). An insight of environmental contamination of arsenic on animal health. Emerging Contaminants, 3(1), 17–22.

[fsn34363-bib-0038] Mengistu, F. G. , Tabor, G. , Dagne, Z. , Atinafu, G. , & Tewolde, F. T. (2021). Effect of planting density on yield and yield components of lettuce (*Lactuca sativa* L.) at two agro‐ecologies of Ethiopia. African Journal of Agricultural Research, 17(4), 549–556.

[fsn34363-bib-0039] Mofokeng, T. P. , Tetana, Z. N. , & Ozoemena, K. I. (2020). Defective 3D nitrogen‐doped carbon nanotube‐carbon fibre networks for high‐performance supercapacitor: Transformative role of nitrogen‐doping from surface‐confined to diffusive kinetics. Carbon, 169, 312–326.

[fsn34363-bib-0040] Musse, Z. A. , Yoseph Samago, T. , & Beshir, H. M. (2020). Effect of liquid bio‐slurry and nitrogen rates on soil physico‐chemical properties and quality of green bean (*Phaseolus vulgaris* L.) at Hawassa Southern Ethiopia. Journal of Plant Interactions, 15(1), 207–212.

[fsn34363-bib-0041] Nasir, I. M. , Mohd Ghazi, T. I. , & Omar, R. (2012). Anaerobic digestion technology in livestock manure treatment for biogas production: A review. Engineering in Life Sciences, 12(3), 258–269.

[fsn34363-bib-0042] Nicolodelli, G. , Senesi, G. S. , de Oliveira Perazzoli, I. L. , Marangoni, B. S. , Benites, V. D. M. , & Milori, D. M. B. P. (2016). Double pulse laser induced breakdown spectroscopy: A potential tool for the analysis of contaminants and macro/micronutrients in organic mineral fertilizers. Science of the Total Environment, 565, 1116–1123. 27261426 10.1016/j.scitotenv.2016.05.153

[fsn34363-bib-0043] Ning, C. C. , Gao, P. D. , Wang, B. Q. , Lin, W. P. , Jiang, N. H. , & Cai, K. Z. (2017). Impacts of chemical fertilizer reduction and organic amendments supplementation on soil nutrient, enzyme activity and heavy metal content. Journal of Integrative Agriculture, 16(8), 1819–1831.

[fsn34363-bib-0044] NMSA . (2020). National Metrology Service Agency (NMSA), Hawassa Branch, Ethiopia.

[fsn34363-bib-0045] Nuhriawangsa, A. M. P. , Ardika, D. , Kartikasari, L. R. , & Hertanto, B. S. (2021). A study on physical characteristics of dried bio‐slurry produced in tropical condition through treatment combination of drying and turning period. In IOP Conference Series: Earth and environmental science (Vol. 902, 012013). IOP Publishing.

[fsn34363-bib-0046] Oliva, M. , Camas, D. E. , Valqui, X. J. , Meléndez, J. B. , & Leiva, S. (2019). Quantitative determination of Cadmium (Cd) in soil‐plant system in potato cropping (*Solanum tuberosum* var. Huayro). Advances in Agriculture, 2019, 1–4.

[fsn34363-bib-0072] Olsen, S. R. , Cole, C. V. , Watanabe, F. S. , & Dean, L. A. (1954). Estimation of available phosphorus in soils by extraction with sodium carbonate (Vol. 939, pp. 1–19). USDA Circular.

[fsn34363-bib-0047] Pahalvi, H. N. , Rafiya, L. , Rashid, S. , Nisar, B. , & Kamili, A. N. (2021). Chemical fertilizers and their impact on soil health. In Microbiota and biofertilizers, ecofriendly tools for reclamation of degraded soil environs (Vol. 2, pp. 1–20). Springer.

[fsn34363-bib-0048] Pirsaheb, M. , Fattahi, N. , Sharafi, K. , Khamotian, R. , & Atafar, Z. (2016). Essential and toxic heavy metals in cereals and agricultural products marketed in Kermanshah, Iran, and human health risk assessment. Food Additives & Contaminants: Part B, 9(1), 15–20. 10.1080/19393210.2015.1099570 26465977

[fsn34363-bib-0049] Qin, J. , Liu, H. , Zhao, J. , Wang, H. , Zhang, H. , Yang, D. , & Zhang, N. (2020). The roles of Bacteria in soil organic carbon accumulation under nitrogen deposition in Stipa baicalensis steppe. Microorganisms, 8(3), 326. 32110984 10.3390/microorganisms8030326 PMC7142556

[fsn34363-bib-0050] Rahaman, M. S. , Rahman, M. M. , Mise, N. , Sikder, M. T. , Ichihara, G. , Uddin, M. K. , Kurasaki, M. , & Ichihara, S. (2021). Environmental arsenic exposure and its contribution to human diseases, toxicity mechanism and management. Environmental Pollution, 289, 117940. 34426183 10.1016/j.envpol.2021.117940

[fsn34363-bib-0051] Rehman, M. Z. , Zafar, M. , Waris, A. A. , Rizwan, M. , Ali, S. , Sabir, M. , Usman, M. , Ayub, M. A. , & Ahmad, Z. (2020). Residual effects of frequently available organic amendments on cadmium bioavailability and accumulation in wheat. Chemosphere, 244, 125548. 32050343 10.1016/j.chemosphere.2019.125548

[fsn34363-bib-0052] Rowell, D. L. (2014). Soil science: Methods & applications. Routledge.

[fsn34363-bib-0053] Sandeep, G. , Vijayalatha, K. R. , & Anitha, T. (2019). Heavy metals and its impact in vegetable crops. International Journal of Chemical Studies, 7(1), 1612–1621.

[fsn34363-bib-0070] SAS . (2011). Base SAS ® 9.3. SAS Institute Inc., Cary, NC, USA.

[fsn34363-bib-0054] Sayara, T. , Basheer‐Salimia, R. , Hawamde, F. , & Sánchez, A. (2020). Recycling of organic wastes through composting: Process performance and compost application in agriculture. Agronomy, 10(11), 1838.

[fsn34363-bib-0055] Shahbaz, M. , Sbia, R. , Hamdi, H. , & Ozturk, I. (2014). Economic growth, electricity consumption, urbanization and environmental degradation relationship in United Arab Emirates. Ecological Indicators, 45, 622–631.

[fsn34363-bib-0056] Shen, B. , Wang, X. , Zhang, Y. , Zhang, M. , Wang, K. , Xie, P. , & Ji, H. (2020). The optimum pH and Eh for simultaneously minimizing bioavailable cadmium and arsenic contents in soils under the organic fertilizer application. Science of the Total Environment, 711, 135229. 32000353 10.1016/j.scitotenv.2019.135229

[fsn34363-bib-0058] Sinduja, M. , Sathya, V. , Maheswari, M. , Kalpana, P. , Dhevagi, P. , Dinesh, G. K. , & Chitdeshwari, T. (2023). Chemical transformation and bioavailability of chromium in the contaminated soil amended with bioamendments. Bioremediation Journal, 27(3), 229–250.

[fsn34363-bib-0071] Tadese, T . (1991). *Soil, plant, water, fertilizer, animal manure and compost analysis* (Working Document No. 13). International Livestock Research Center for Africa, Addis Ababa.

[fsn34363-bib-0060] Tegegne, W. A. (2015). Assessment of some heavy metals concentration in selected cereals collected from local markets of Ambo City, Ethiopia. Journal of Cereals and Oilseeds, 6(2), 8–13.

[fsn34363-bib-0061] Tekle, L. , Abebe, N. , & Asfaw, M. (2018). On farm demonstration of improved lettuce variety (*Lactuca sativa*) in Southeastern zone of Tigray, Ethiopia. Journal of Plant Breeding and Crop Science, 10(7), 178–182.

[fsn34363-bib-0063] Ubuoh, E. A. , Umezuruike, S. O. , Nworuh, B. O. , & Emeka, C. C. (2019). Assessment of soil pH and heavy metal concentrations in agricultural land impacted with medical waste incinerator (MWI) flue ash (FA) in Abia State, Nigeria. Journal of Applied Sciences and Environmental Management, 23(2), 275–282.

[fsn34363-bib-0064] Wang, B. , Liu, C. , Chen, Y. , Dong, F. , Chen, S. , Zhang, D. , & Zhu, J. (2018). Structural characteristics, analytical techniques and interactions with organic contaminants of dissolved organic matter derived from crop straw: A critical review. RSC Advances, 8(64), 36927–36938. 35558903 10.1039/c8ra06978f PMC9089241

[fsn34363-bib-0065] Wang, Z. , Sanusi, I. A. , Wang, J. , Ye, X. , Kana, E. B. G. , Olaniran, A. O. , & Shao, H. (2023). Developments and prospects of farmland application of biogas slurry in China—A review. Microorganisms, 11(11), 2675. 38004687 10.3390/microorganisms11112675 PMC10673569

[fsn34363-bib-0074] Wuana, R. A. , & Okieimen, F. E. (2011). Heavy metals in contaminated soils: a review of sources, chemistry, risks and best available strategies for remediation. International Scholarly Research Notices, 2011(1), 402647.

[fsn34363-bib-0066] Yeshiwas, Y. (2017). Effect of different rate of nitrogen fertilizer on the growth and yield of cabbage (*Brassica oleraceae*) at Debre Markos, North West Ethiopia. African Journal of Plant Science, 11(7), 276–281.

[fsn34363-bib-0067] Zemichael, B. , Hadush, M. , & Abebe, N. (2017). Effect of inter and intra‐row spacing on yield and yield components of lettuce (*Lactuca sativa*) in South East Tigray, Ethiopia. Biomedical Journal of Scientific & Technical Research, 1(6), 1698–1701.

[fsn34363-bib-0068] Zhang, S. , Song, J. , Cheng, Y. , & Lv, M. (2018). Proper management of lead‐contaminated agricultural lands against the exceedance of lead in agricultural produce: Derivation of local soil criteria. Science of the Total Environment, 634, 321–330. 29627556 10.1016/j.scitotenv.2018.03.337

[fsn34363-bib-0069] Zhen, H. , Jia, L. , Huang, C. , Qiao, Y. , Li, J. , Li, H. , Chen, Q. , & Wan, Y. (2020). Long‐term effects of intensive application of manure on heavy metal pollution risk in protected‐field vegetable production. Environmental Pollution, 263, 114552. 32305799 10.1016/j.envpol.2020.114552

